# Presence of *Burkholderia pseudomallei* in Soil and Paddy Rice Water in a Rice Field in Northeast Thailand, but Not in Air and Rainwater

**DOI:** 10.4269/ajtmh.17-0515

**Published:** 2017-10-02

**Authors:** Catherine E. L. Ong, Gumphol Wongsuvan, Janet S. W. Chew, Tan Yian Kim, Low Hwee Teng, Premjit Amornchai, Vanaporn Wuthiekanun, Nicholas P. J. Day, Sharon J. Peacock, Tan Yoke Cheng, Eric P. H. Yap, Direk Limmathurotsakul

**Affiliations:** 1 Defence Medical and Environmental Research Institute, DSO National Laboratories, Singapore, Singapore;; 2 Mahidol-Oxford Tropical Medicine Research Unit, Faculty of Tropical Medicine, Mahidol University, Bangkok, Thailand;; 3 Centre for Tropical Medicine and Global Health, Nuffield Department of Medicine, University of Oxford, Oxford, United Kingdom;; 4 Department of Microbiology and Immunology, Faculty of Tropical Medicine, Mahidol University, Bangkok, Thailand;; 5 Department of Medicine, Cambridge University, Addenbrooke’s Hospital, Cambridge, United Kingdom;; 6 Lee Kong Chian School of Medicine, Nanyang Technological University, Singapore;; 7 Department of Tropical Hygiene, Faculty of Tropical Medicine, Mahidol University, Bangkok, Thailand

## Abstract

Environmental *Burkholderia pseudomallei* has been postulated to be aerosolized during ploughing and heavy rain, and could result in inhalational melioidosis. Here, we determined the presence of *B. pseudomallei* in soil, paddy field water (PFW), air, and rainwater samples in a single rice paddy field in Ubon Ratchathani, northeast Thailand. In 2012, we collected 100 soil samples during the dry season, 10 PFW samples during the monsoon season, 77 air samples during ploughing (*N* = 31) and heavy rains (*N* = 46), and 60 rainwater samples during 12 rain events. We found that 32 soil samples (32%), six PFW samples (60%), and none of the air and rainwater samples were culture positive for *B. pseudomallei*. Other soil bacteria were isolated from air and rainwater samples. Mean quantitative count of *B. pseudomallei* estimated from two culture-positive PFW samples was 200 colony forming units/mL. Our findings suggest that the risk of melioidosis acquisition by inhalation in Thailand might be low.

*Burkholderia pseudomallei*, the causative agent of melioidosis, is commonly found in soil and groundwater in many tropical countries worldwide.^1^ The bacterium is intrinsically resistant to a wide range of antimicrobials, and treatment with ineffective antimicrobials may result in case fatality rates exceeding 70%.^2,3^ A recent modeling study estimated that there are about 165,000 human melioidosis cases per year globally, and 89,000 of them (54%) die.^1^ Skin inoculation is considered the main route of infection in farmers working in rice paddy fields in Thailand.^4^ Presence of *B. pseudomallei* in the environment indicates an area at risk for the acquisition of melioidosis.^5,6^ Recent evidence also suggests that ingestion of *B. pseudomallei* contaminated water,^7^ and inhalation of *B. pseudomallei* during extreme weather events^8–10^ are also important routes of infection.

In this study, we conducted environmental sampling to determine the presence of *B. pseudomallei* in soil, paddy field water (PFW), air and rainwater samples at a single rice paddy field located at Amphoe Lao Suea Kok, Ubon Ratchathani, northeast Thailand. The study was conducted during both the dry season (March) and monsoon season (August) in 2012. This study site was previously determined to be culture positive for *B. pseudomallei* from soil samples.^11^

During the dry season in March, we collected 100 soil samples and 31 air samples (Table 1). The paddy field was dry with sandy loam soil. For soil samples, we used the consensus guidelines for environmental sampling described by the Detection of Environmental *Burkholderia pseudomallei* Working Party.^5^ In brief, the rice field was divided into a grid system, in which 100 sampling points (10 by 10) were plotted 2.5 m apart. At each sampling point, around 10 g of soil was removed from the base of a 30-cm hole. The soil samples were mixed with 10 mL of enrichment broth consisting of threonine-basal salt solution plus colistin (TBSS-C50 broth) and incubated at 40°C in air for 48 hours. Ten microliters of surface liquid was then subcultured onto Ashdown agar, incubated at 40°C in air, and examined daily for 4 days for bacterial colonies suggestive of *B. pseudomallei*, which were initially identified on the basis of colony morphotype.^12^ This included the characteristic colony morphology (purple, flat, dry, and wrinkled) together with six additional colony morphotypes, as described previously.^12^ Presumptive colonies were picked from each sample and tested immediately using a specific latex agglutination test for *B. pseudomallei-*specific capsular polysaccharide^13^ and the immunofluorescence assay,^14^ as previously described. For air sampling, three types of air samplers with different airflow volumes and collection media were used during different activities in the same field. Using a mobile meteorological station (Davis Weather Station), we measured the wind speed (1.0–2.4 m/s) and established the wind direction, which was mainly from South-West during the dry season sampling (Figure 1). The Casella Slit Agar Sampler (single slit, air flow volume 300 L/min, Ashdown agar medium plate collection, Casella, US) and XMX 2L-MIL Bioaerosol Collector (700 L/min, phosphate-buffered saline (PBS) collection medium, Dycor Technologies, Canada) were established at a sampling height of 1.5 m above ground level, downwind of the sampling activities to simulate the height achieved by human inhalation. Four SKC Air Samplers (5 L/min, 0.2-μm polycarbonate membrane filter; SKC Inc.) were placed on tripods at four corners of the field. An additional SKC Air Sampler was carried by a researcher during soil digging and ploughing performed by farmers and a tractor, respectively. First, air sampling was conducted when there was no activity to determine whether *B. pseudomallei* could be isolated from the air in the absence of human activities (*N* = 7). Thereafter, air sampling was conducted during soil digging (*N* = 15) and during tractor ploughing in the field (*N* = 9). Generous dust cloud was observed while the tractor was ploughing. The 140-mm Ashdown agar plates used for Casella Slit Agar Sampler were incubated at 40°C for 48 hours before reading. Samples were processed using the direct plating method or enrichment method for each of the 5-mL PBS samples collected from the XMX-2L MIL Bioaerosol Collector. Briefly, 10 μL and 100 μL of PBS sample were plated onto Ashdown agar plates directly in replicates and incubated at 40°C for 4 days. Another 1 mL of PBS was aliquoted into 9 mL TBSS-C50 broth, vortexed vigorously and incubated at 40°C for 48 hours, after which 10 μL was subcultured onto Ashdown agar plates in replicates and processed similarly to the direct plating method. All plates were observed daily for 4 days for presence of *B. pseudomallei* colonies. For each SKC unit, the filter was transferred into 10-mL TBSS-C50 broth, vortexed vigorously, and incubated at 40°C for 48 hours. Thereafter, the samples were processed similarly as per the enrichment method.

**Table 1:** Environmental samples collected in a single rice field in Ubon Ratchathani

Samples (*n*)	Season (Date)	Activity	Air samplers used (*n*)	No. of samples culture positive for *B. pseudomallei*
Soil (100 samples)	Dry (March 5)	None (baseline)	None (*N* = 100)	32 (32%)
Air (31 samples)	Dry (March 5)	None (baseline)	Casella (*N* = 1), XMX (*N* = 1), SMC (*N* = 5)	0
	Dry (March 5)	Soil digging	Casella (*N* = 5), XMX (*N* = 5), SMC (*N* = 5)	0
	Dry (March 5)	Tractor ploughing	Casella (*N* = 2), XMX (*N* = 2), SMC (*N* = 5)	0
Paddy field water (10 samples)	Rainy (August 20)	None (flooded rice field)	None (*N* = 5)	2*
	Rainy (August 24)	None (flooded rice field)	None (*N* = 5)	4†
Rainwater (60 samples)	Rainy (August 2)	Raining during the day	None (*N* = 5)	0
	Rainy (August 3)	Raining during the day	None (*N* = 5)	0
	Rainy (August 5)	Raining during the night	None (*N* = 5)	0
	Rainy (August 7)	Raining during the night	None (*N* = 5)	0
	Rainy (August 9)	Raining during the night	None (*N* = 5)	0
	Rainy (August 11)	Raining during the night	None (*N* = 5)‡	0
	Rainy (August 15)	Raining during the night	None (*N* = 5)‡	0
	Rainy (August 16)	Raining during the night	None (*N* = 5)‡	0
	Rainy (August 17)	Raining during the day	None (*N* = 5)‡	0
	Rainy (August 17)	Raining during the night	None (*N* = 5)‡	0
	Rainy (August 18)	Raining during the night	None (*N* = 5)‡	0
	Rainy (August 19)	Raining during the night	None (*N* = 5)‡	0
Air (46 samples)	Rainy (August 2)	Raining during the day	Casella (*N* = 1)‡, XMX (*N* = 4)	0
	Rainy (August 3)	Raining during the day	Casella (*N* = 1)‡, XMX (*N* = 5)	0
	Rainy (August 12)	None (baseline, no raining)	Casella (*N* = 4), XMX (*N* = 8)	0
	Rainy (August 14)	Light drizzle during the day	Casella (*N* = 2), XMX (*N* = 2)	0
	Rainy (August 15)	None (baseline, no raining)	Casella (*N* = 2), XMX (*N* = 4)‡	0
	Rainy (August 16)	After overnight rain	Casella (*N* = 2)‡, XMX (*N* = 4)	0
	Rainy (August 17)	After overnight rain	Casella (*N* = 1)‡	0
	Rainy (August 18)	Raining during the day	Casella (*N* = 2), XMX (*N* = 4)	0

* Quantitative count of *B. pseudomallei* was 150 and 250 CFU/mL.

† Quantitative count of *B. pseudomallei* was not performed.

‡ *Burkholderia pseudomallei-*like bacterial colonies were observed.

**Figure 1.**
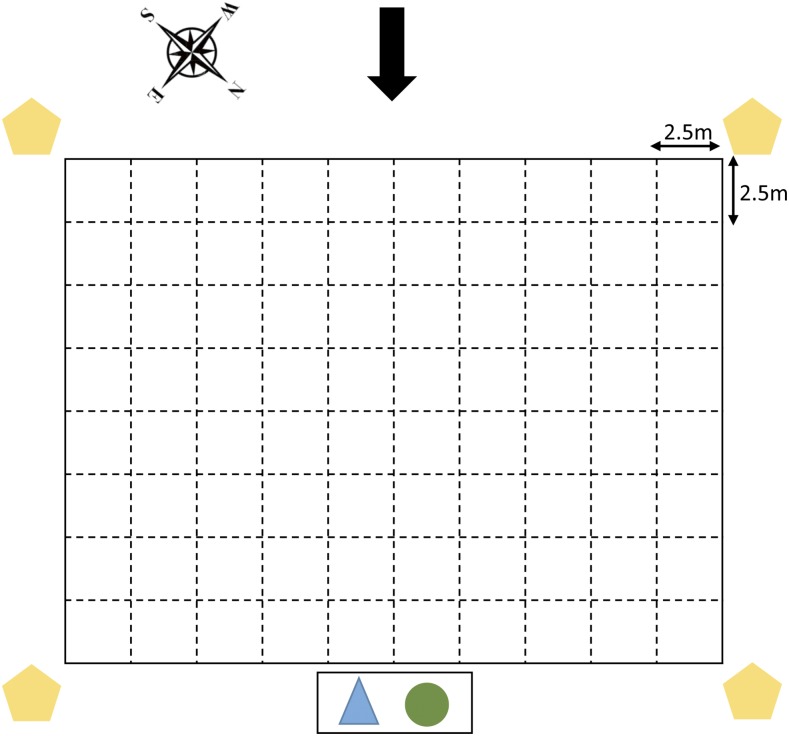
Schematic diagram showing the sampled field, wind direction, and locations of air samplers during the dry season. The presence of *Burkholderia pseudomallei* in 100 equally spaced sampling points measuring 2.5 m by 2.5 m in a rice paddy field in Ubon Ratchathani was investigated during the dry season. The center point of each square was sampled for soil. The black arrow (top) indicates the direction of the prevailing wind. Orange pentagons represent the positions of SKC Air Samplers, the blue triangle represents the location of the Casella Slit Agar Sampler, and the green circle represents the location of the XMX 2L-MIL Bioaerosol Collector. This figure appears in color at www.ajtmh.org.

During the monsoon season in August, we revisited the same site. We collected PFW, air, and rainwater samples and cultured these for *B. pseudomallei.* PFW samples were collected in 50-mL Falcon tubes by opening the cap, after submerging the tube into the water to a depth of about 10 cm, in flooded rice paddy field. A total of 10 samples were collected from five different locations in the rice field on two separate days. 10 μL of PFW was plated on Ashdown agar plates in replicates, and colony counts were conducted as previously described.^15^ We also conducted air sampling when there were no human activities, when it was raining, and in the morning after overnight rain. Air samplers were located in the middle of the sampling area to take account of variable wind direction. We collected a total of 15 air samples using the Casella Slit Agar Sampler and 31 air samples with the XMX-2L MIL Bioaerosol Collector (Table 1). Rainwater was collected in clean plastic bag-lined collection pails (12″ diameter) supported on a tall stool approximately 130 cm above the ground to minimize potential contamination from soil particles splashed up during heavy rainfall. A total of 12 rain events occurred during our study, with many of the heavy rains occurring late at night (Table 1). The rainwater collected from day events was transported back to the laboratory and processed on the same day whereas those collected from overnight rain events was processed accordingly on the following day. Each rainwater sample was filtered and concentrated using a custom-developed water filter machine using diatomaceous earth and nitrocellulose membranes. The diatomaceous earth containing trapped particles from the filtered rainwater was transferred into 50-mL Falcon tube with 20-mL trypticase soy broth plus crystal violet at 5 mg/L and colistin at 50 mg/L, and incubated at 40° for 48 hours. Thereafter, 10 μL of sample was inoculated onto Ashdown agar and further incubated for 4 days. All culture plates were subsequently examined for the presence of suspected *B. pseudomallei* colonies.

We found that 32 of 100 (32%) soil samples and none of the 31 air samples collected during the dry season were culture positive for *B. pseudomallei* (Table 1). The culture plates from the Casella air sampler, collected during tractor ploughing (*N* = 2), were unidentified bacterial colonies with an overgrowth of fungus, indicating that bacterial and fungal spores were aerosolized during the ploughing. The other air samples had no organisms isolated.

During the monsoon season, six of ten (60%) PFW samples, and none of the 46 air samples and 60 rain water samples were culture positive for *B. pseudomallei* (Table 1). Quantitative counts of *B. pseudomallei* were performed in the first two culture-positive PFW samples and were 150 and 250 colony forming units (CFU)/mL (mean 200 CFU/mL). Qualititative culture was performed for the later four culture-positive PFW samples. We obtained many bacterial colonies with *B. pseudomallei*-like colony morphology from rainwater samples and air samples (from both Casella and XMX air samplers; Table 1). All isolates were negative by latex agglutination and by indirect immunofluorescent assay. We randomly selected nine isolates with different colony morphologies (four from air samples and five from rainwater samples) (Figure 2) and evaluated their identities by using DNA extraction and 16S rDNA characterization.^16^ Subsequent blasting identified common soil bacteria, including *Sphingobacterium thalpophilum* (*N* = 1), *Stenotrophomonas pavanii* (*N* = 1), *Delftia lacustris/tsuruhatensis* (*N* = 1), and *Cupriavidus necator/brasiliensis* (*N* = 1) from air samples, and *Ralstonia picketii* (*N* = 2) and members from the *Burkholderia cepacia* complex, including *Burkholderia latens* (*N* = 1), *Burkholderia multivorans* (*N* = 1), and *Burkholderia ambifaria* (*N* = 1) from rainwater samples.

**Figure 2.**
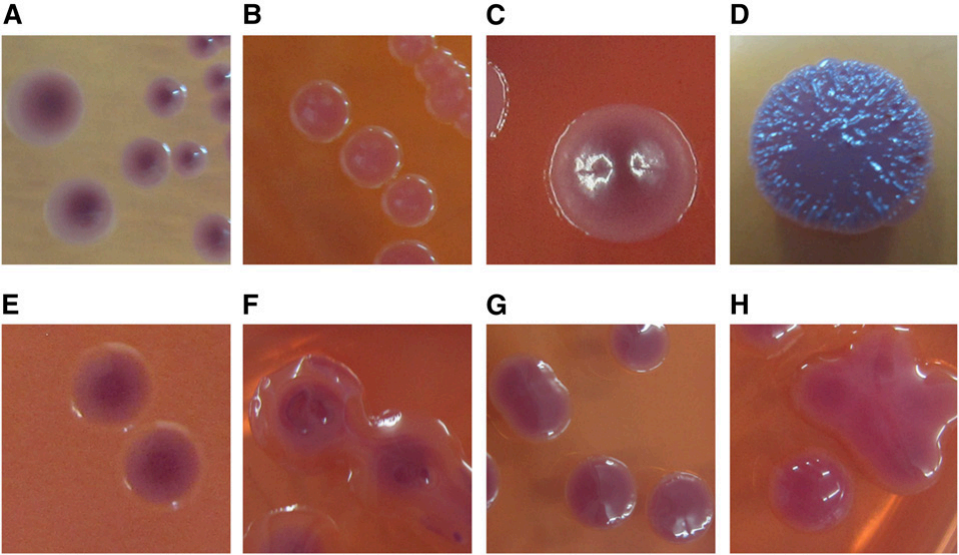
Eight organisms with *Burkholderia pseudomallei*-like bacterial colonies isolated from air and rainwater samples in Ubon Ratchathani, Thailand. Bacterial colonies isolated from air samples using Casella Slit Agar Sampler: (**A**) *Sphingobacterium thalpophilum*, (**B**) *Stenotrophomonas pavanii*, (**C**) *Delftia lacustris/tsuruhatensis,* and using the XMX-MIL BioAerosol Collector: (**D**) *Cupriavidus necator/brasiliensis*. Bacterial colonies isolated from rainwater: (**E**) *Ralstonia pickettii*, (**F**) *Burkholderia latens*, (**G**) *Burkholderia multivorans*, and (**H**) *Burkholderia ambifaria*. This figure appears in color at www.ajtmh.org.

Our findings suggest that the risk of melioidosis acquisition by inhalation in Thailand might be low and that the main route of disease acquisition is skin inoculation due to occupational exposure to soil and water in paddy fields. The relative ease of isolation of *B. pseudomallei* from PFW supports an increased risk of exposure to the bacterium in a flooded paddy rice field.^4^ The traditional culture of rice planting with bare hands and exposed feet for long hours also increases the risk of infection.^4^ It is thus imperative to continue to educate farmers on the need to wear protective gear such as rubber gloves and boots or waders during rice planting to prevent melioidosis infections.^4,17^

Despite failure to isolate *B. pseudomallei* from air and rainwater samples, our findings confirm that soil bacteria can be suspended in the air, particularly during and after rain and that rainwater is commonly contaminated with soil bacteria. Our findings do not rule out the possibility of *B. pseudomallei* inhalation in Thailand. Failure of isolation could be due to the low concentration of *B. pseudomallei* in the air during our study period. It is also possible that the wind speed during the monsoon season in Thailand is comparatively lower than those observed in Hong Kong,^18^ Taiwan^8^ and Darwin (Northern Territory, Australia)^10^ where *B. pseudomallei* has been isolated from the air. The total number of culture-confirmed melioidosis patients presenting to Sunpasitthiprasong Hospital, Ubon Ratchathani was around 400 cases per year between 2007 and 2015 (Supplemental Table 1). Further studies are required to understand the route of *B. pseudomallei* acquisition in individual patients. We continue to support the suggestion that people in melioidosis-endemic areas, including Thailand, should avoid heavy rain or dust clouds and should only drink bottled or boiled water.^4^

## Supplementary Material

Supplemental Table.
