# Supplementary material for: Presence of Burkholderia pseudomallei in Soil and Paddy Rice Water in a Rice Field in Northeast Thailand, but Not in Air and Rainwater
Source: Am J Trop Med Hyg. 2017 Oct 2;97(6):1702–5. doi: 10.4269/ajtmh.17-0515 (PMC5805070; doi:10.4269/ajtmh.17-0515)
Supplement: Supplementary file 1 [file tpmd170515.SD1.pdf]

Supplemental Table 1

Incidence of melioidosis and mortality from 2007 to 2015 at Sunpasitthiprasong Hospital, Ubon Ratchathani, northeast Thailand*

Year	Melioidosis patients (*N*)†	Deaths	Mortality (%)
2007	475	167	35
2008	401	107	27
2009	383	119	31
2010	393	140	36
2011	405	143	35
2012	405	157	39
2013	450	199	44
2014	444	174	39
2015	378	134	35

*Incidence of melioidosis and mortality from 1997 to 2006 at Sunpasithiprasong Hospital was previously reported.[19]

† Diagnosed by growth of *B. pseudomallei* from any clinical specimen.

SUPPLEMENTAL REFERENCE

19. Limmathurotsakul D, Wongratanacheewin S, Teerawattanasook N, Wongsuvan G, Chaisuksant S, Chetchotisakd P, Chaowagul W, Day NP, Peacock SJ, 2010. Increasing incidence of human melioidosis in northeast Thailand. *Am J Trop Med Hyg 82:* 1113–1117.
